# Reducing New Chlamydia Infection Among Young Men by Promoting Correct and Consistent Condom Use: Protocol for a Randomized Controlled Trial

**DOI:** 10.2196/35729

**Published:** 2022-08-10

**Authors:** Nicole Stone, Rowena Bedford, Katie Newby, Katherine Brown, Louise Jackson, Stephen Bremner, Leanne Morrison, Nuala McGrath, Tom Nadarzynski, Jake Bayley, Nicky Perry, Cynthia Graham

**Affiliations:** 1 Department of Psychology Faculty of Environmental and Life Sciences University of Southampton Southampton United Kingdom; 2 Department of Psychology and Sports Science Faculty of Life and Medical Sciences University of Hertfordshire Hertfordshire United Kingdom; 3 Institute of Applied Health Research College of Medical and Dental Sciences University of Birmingham Birmingham United Kingdom; 4 Department of Primary Care and Public Health Brighton and Sussex Medical School Brighton United Kingdom; 5 School of Primary Care, Population Sciences and Medical Education Faculty of Medicine University of Southampton Southampton United Kingdom; 6 Department of Social Statistics and Demography Faculty of Economic, Social and Political Sciences University of Southampton Southampton United Kingdom; 7 School of Social Sciences University of Westminster London United Kingdom; 8 Barts Health NHS Trust London United Kingdom; 9 University Hospitals Sussex NHS Trust Brighton United Kingdom

**Keywords:** condom fit and feel, condom use, pleasure, digital intervention, sexual behavior, health psychology, behavior intervention, chlamydia, sexual health, randomized controlled trial

## Abstract

**Background:**

The health, social, and economic costs of sexually transmitted infections (STIs) represent a major public health concern. Young people are considered one of the groups most at risk for acquiring and transmitting STIs. Correct and consistent condom use has been shown to be the most effective method for reducing STIs; however, condoms are often not used properly. Evidence shows that brief behavior change interventions that focus on skills, communication, and motivation to acquire safe sex practices should be adopted into routine care to reduce STIs. Funding for sexual health services in England has declined dramatically, so novel ways of reducing clinic attendance are being sought. The home-based intervention strategy (HIS-UK) to promote condom use among young men has shown promise in feasibility and pilot studies by demonstrating high acceptability of the intervention in participant and health professional feedback, including aiding men to find condoms they like and feel more confident when using condoms.

**Objective:**

The aim of this study is to determine the effectiveness and cost-effectiveness of HIS-UK when compared to usual condom distribution care among young men.

**Methods:**

The 3 trial arms consisting of “e-HIS” (HIS-UK delivered digitally), “ProHIS” (HIS-UK delivered face-to-face), and control condition (usual National Health Service [NHS] care) will be compared against the following 3 primary outcomes: the extent to which correct and consistent condom use is increased; improvement of condom use experiences (pleasure as well as fit and feel); and decrease in chlamydia test positivity. Eligibility criteria include men aged 16-25 years at risk of STIs through reporting of condom use errors (ie, breakage or slippage) or condomless penile-vaginal or penile-anal intercourse with casual or new sexual partners during the previous 3 months. Prospective participants will be recruited through targeted advertisements and an opportunistic direct approach at selected sexual health and genitourinary medicine services and university-associated health centers and general practitioner practices. Community and educational establishments will be used to further advertise the study and signpost men to recruitment sites. Participants will be randomly allocated to 1 of 3 trial arms. A repeated measures design will assess the parallel arms with baseline and 12 monthly follow-up questionnaires after intervention and 3 chlamydia screening points (baseline, 6, and 12 months).

**Results:**

Recruitment commenced in March 2020. Due to the COVID-19 pandemic, the study was halted and has since reopened for recruitment in Summer 2021. A 30-month recruitment period is planned.

**Conclusions:**

If effective and cost-effective, HIS-UK can be scaled up into routine NHS usual care to reduce both STI transmission in young people and pressure on NHS resources. This intervention may further encourage sexual health services to adopt digital technologies, allowing for them to become more widely available to young people while decreasing health inequalities and fear of stigmatization.

**Trial Registration:**

ISRCTN Registry ISRCTN11400820; https://www.isrctn.com/ISRCTN11400820

## Introduction

### Background

Sexually transmitted infections (STIs) are a major public health concern. Individuals affected by STIs can face poor physical and psychological outcomes, and STI testing and treatment are a costly burden on limited health care service budgets [1,2]. The UK Department of Health and Social Care has prioritized the need to reduce STIs among those at greatest risk, including Black and minority ethnic populations, young heterosexual men and women, men who have sex with men, and among those residing in the most deprived areas in England [1,3]. Furthermore, the World Health Organization, the UK Department of Health and Social Care, and Public Health England all recommend that evidence-based preventative interventions should be used in primary care settings to help in the reduction of STI rates [4-6].

Male condoms, when used correctly and consistently (used from the start to the finish of every sex act), are highly effective in preventing STI transmission [7,8]. While being highly effective, there is substantial evidence to indicate that condoms are not used frequently, and if they are, they are often not used properly [9-11]. Research has shown there are several common errors and problems associated with condom usage, including not checking the condom for visible damage, not leaving room at the tip of the condom, and using oil-based lubricants, which could be detrimental to the condom material [10,12,13].

Behavior change intervention programs to promote condom use typically aim to improve knowledge and skills, reduce communication barriers, and improve partner negotiation. Research shows, however, that negative condom attitudes form a significant barrier to usage, often shaped through experience of decreased sensation and sexual pleasure, as well as erection difficulties during condom application and use [9]. Many interventions to date have failed to consider the promotion of pleasurable condom use and to address issues around the fit and feel of condoms, including condom size, texture, and thickness. One review found only 5 studies (out of the 123 condom promotion studies identified) focused on improving fit and feel [14], despite its positive association with use [15].

### Intervention

**Figure 1:**
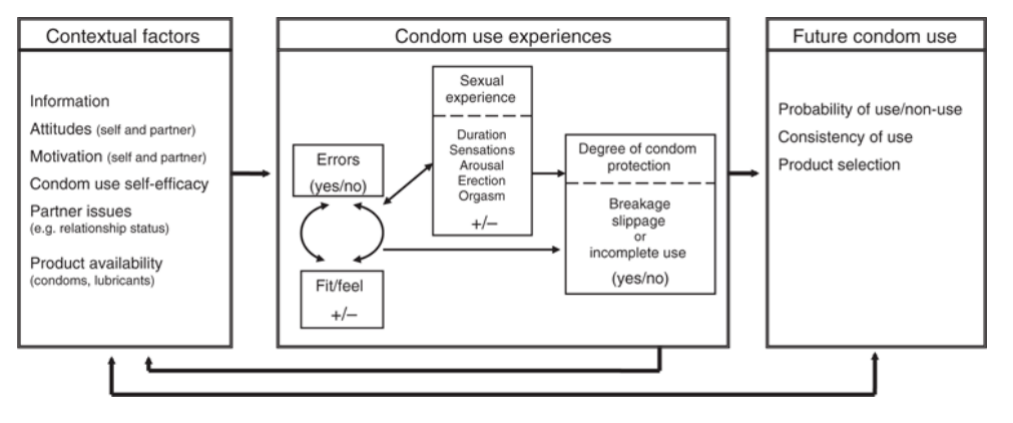
The Condom Use Experience Model (Sanders et al [13], 2012).

A review of the evidence on safe-sex advice underlined the need for novel brief behavior change interventions focusing on skills acquisition, motivation (via affective or automatic attitudinal cognitive processes), and communication competencies to ensure the adoption of safer sexual behaviors [16]. Furthermore, the National Institute for Health and Care Excellence has emphasized the need to provide a range of condoms and lubricants when teaching young people to use condoms effectively and safely [17], with the National Health Service (NHS) of England also highlighting the importance of using digital technologies to deliver health care to help reduce costs, accessibility barriers, and clinical burden [18]. With these recommendations in mind, the home-based intervention strategy (HIS-UK) has been adapted from an intervention previously piloted in the US and Canada (The Kinsey Institute Homework Intervention Strategy [19,20]), guided by the condom use experience (CUE) model proposed by Sanders et al [13]. The CUE model acts as a framework for understanding the role of errors and problems in inadequate condom protection (Figure 1). In the model, contextual factors (eg, knowledge, skills, and self-efficacy), in conjunction with condom use experience (including issues of fit and feel as well as condom use errors and problems), affect the probability and consistency of future condom use either directly or as mediated through other aspects of the sexual experience, such as sensations, discomfort, and duration or intensity of intercourse. The model is dynamic, as the quality of the CUE (past and present) cyclically affects condom-related contextual factors during subsequent sexual encounters, which in turn affect CUE.

The HIS-UK intervention is novel in that it aims to increase condom use by enhancing the fit and feel of condoms and, thereby, increasing outcome expectancies related to enjoyment of sex while using condoms [21]. HIS-UK places the impetus for behavior change on the individual using home-based solitary condom and lubricant practice exercises (“homework”) with focus on pleasurable use [22]. It comprises the following three key elements: (1) “Education & Training”—providing guidance on pleasurable condom use, the variety and types of condoms available (shape, size, texture, and material), how to find the best condom for optimal fit and feel, and the added benefits of using lubricant to sexual enjoyment; (2) “Practice & Experimentation”—the provision of a condom kit containing a wide variety of condoms and lubricants and home-based directed practice exercises in applying, using (masturbating with the use of additional lubricant), and removing condoms in “low pressure” situations (ie, not in the presence of a sexual partner); and (3) “Reflection”: the completion of web-based rating forms about the individual’s experience of the condoms and lubricants tested.

HIS-UK has been designed as an extension to the usual condom distribution care model currently practiced by health promotion professionals, which typically comprises the delivery of safe sex messages, a condom application demonstration, and the supply of generic condoms.

Two development and feasibility studies were conducted to inform the adapted design of the HIS-UK intervention, research methodology, and data collection tools [22-24]. The following 2 delivery models of the education and training component of HIS-UK were designed: (1) face-to-face delivery by a trained health professional (ProHIS) and (2) a digital intervention using an interactive website (e-HIS) and without the need for specialist provider contact. The first of the feasibility studies tested the viability, operability, and acceptability of the ProHIS version of the HIS-UK intervention with men aged 16-25 years. The second study tested the feasibility of e-HIS with men aged 18-69. The findings from both studies indicated the intervention was acceptable to men and health promotion professionals, and the research design, evaluative tools, and outcome measures were appropriate.

### Objective

The aim of this trial is to assess the effectiveness and cost-effectiveness of HIS-UK delivered by the 2 intervention delivery models (ProHIS and e-HIS) to reduce chlamydia test positivity among men aged 16-25 years by enhancing condom use experiences and improving correct and consistent condom use, as compared to usual NHS condom distribution care. Intervention implementation, usability, acceptability, as well as mechanisms for impact will be assessed using a mixed-methods approach guided by our logic model and the intervention evaluation framework proposed by Saunders et al [25].

## Methods

### Trial Design

The HIS-UK study is a randomized, controlled, superiority trial with 3 parallel arms (2 intervention and 1 control arm, with a 1:1:1 allocation), with an internal pilot. A repeated measures trial design is being used with baseline measurement (T0) and monthly follow-up questionnaires (T1-12) after randomization and 3 STI screening points for chlamydia (T0, T6, and T12). Cost and outcome data are being collected to compare the resource use and cost-effectiveness of the 2 HIS-UK delivery models compared to usual condom distribution care. The protocol for this trial was registered with the ISRCTN Clinical Trials Registry in October 2019.

### Recruitment and Participants

The trial is multicentered, delivered from NHS Trust sites across England. Participants are recruited through opportunistic direct approach and patient identification by trained site staff at integrated sexual health and genitourinary medicine services located within the participating Trust sites. Targeted advertising in sixth form colleges and youth advisory services and via social media platforms (eg, Twitter, Instagram, and Facebook) is also being used.

Eligible participants must have a penis, be aged 16-25 years, resident in England, and at risk of STIs through reporting either condom use errors (ie, breakage or slippage) or condomless penile-vaginal or penile-anal intercourse with casual (nonregular) or new sexual partners during the previous 3 months. Those with a recognized latex allergy, restricted internet access, or limited proficiency in spoken English are excluded from the trial.

### Baseline Data Collection and Randomization

The trial is delivered using *Lifeguide*, an interactive web-based intervention software platform and secure validated data management system designed to collect participant information and deliver digital interventions to support health behavior change [23,26]. The *Lifeguide* platform can be accessed via any internet-enabled device, and recruitment sites are provided with handheld tablet computers for recruitment purposes.

Following eligibility screening and the taking of informed online consent, participants are registered for the trial and complete a baseline questionnaire (T0). At baseline, participants are asked about their sexual behavior, condom attitudes, condom usage experiences, STI screening, demographics, quality of life (using standard measures), and any recent NHS and public sector resource use (ie, attendance at general practitioner clinics; see Secondary Process Indicators for further details). On submission of T0, participants are randomized to a trial arm (1:1:1) by an in-built *Lifeguide* algorithm to eliminate direct exposure of the allocation process by any members of the research team or recruitment site staff. The algorithm uses randomly permuted blocks of varying length to preserve concealment and maintain balance, with stratification by participant ethnicity and sexual risk. Site staff are informed of the randomization outcome (allocation either to ProHIS, e-HIS, or control arm) by an on-screen notification.

Participants randomized to the ProHIS arm receive a HIS-UK education and training consultation delivered by a health professional (approximately 10 minutes), and participants allocated to receive e-HIS are provided with log-in access to the e-HIS education and training website for them to access at their own leisure. The webpages of e-HIS are delivered and managed by *Lifeguide* using a series of intelligent “agents” (interaction, information, instruction, and evaluation). The purpose of the agents is to manage and monitor the learning of individuals by observing and recording e-learning behavior (ie, pages visited and instructional videos watched) to receive tailored prompts (through automated texts or emails) that guide and assist effective learning (eg, to visit further pages and to undertake tasks) and to ensure participants are exposed to a variety of learning elements.

**Figure 2:**
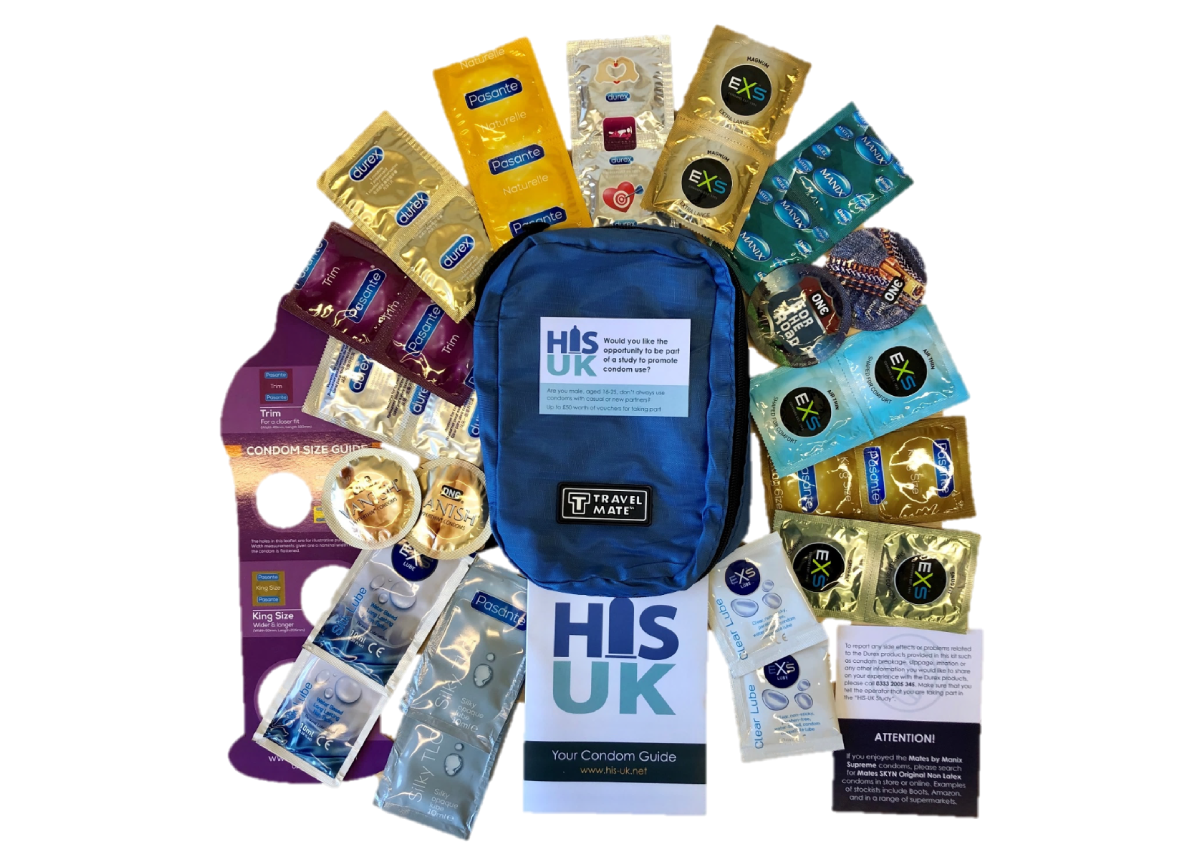
Contents of the home-based intervention strategy (HIS-UK) condom kit.

HIS-UK participants (both ProHIS and e-HIS) are also provided with a condom kit containing 24 condoms (at least 8 different types, shapes, and sizes to demonstrate the wide range of condoms available), 12 lubricant sachets (at least 3 different types), a condom-measuring guide, and an instruction guide to home-based self-practice (Figure 2).

Participants randomized to the control arm receive information, advice, and supplies per usual condom distribution care (eg, per usual care at the recruitment site).

Individual trial participants are not blinded in the trial. Site staff are required to deliver all intervention arms and, as such, are also not blinded. To avoid bias and potential contamination between trial arms, in a 7% random selection of cases, the participant-staff interaction will be audio taped (with participant consent) and assessed for intervention fidelity. In addition, in all cases where ProHIS and usual care are delivered, participants are asked to complete a postintervention fidelity checklist and recall the topics covered during their intervention consultations.

At the baseline visit, participants are further required to undertake screening for chlamydia (the most commonly occurring STI among young men), as per usual clinical practice; a single urine sample is requested from men who report only sexual contact with women, and a triple test (urine sample, anal swab, and oral swab) from men who report sex with men. Sites are responsible for the collection and laboratory analysis of samples, and screening results are subsequently shared by site staff with the research team via the *Lifeguide* platform.

### Post–COVID-19 Amendments

**Figure 3:**
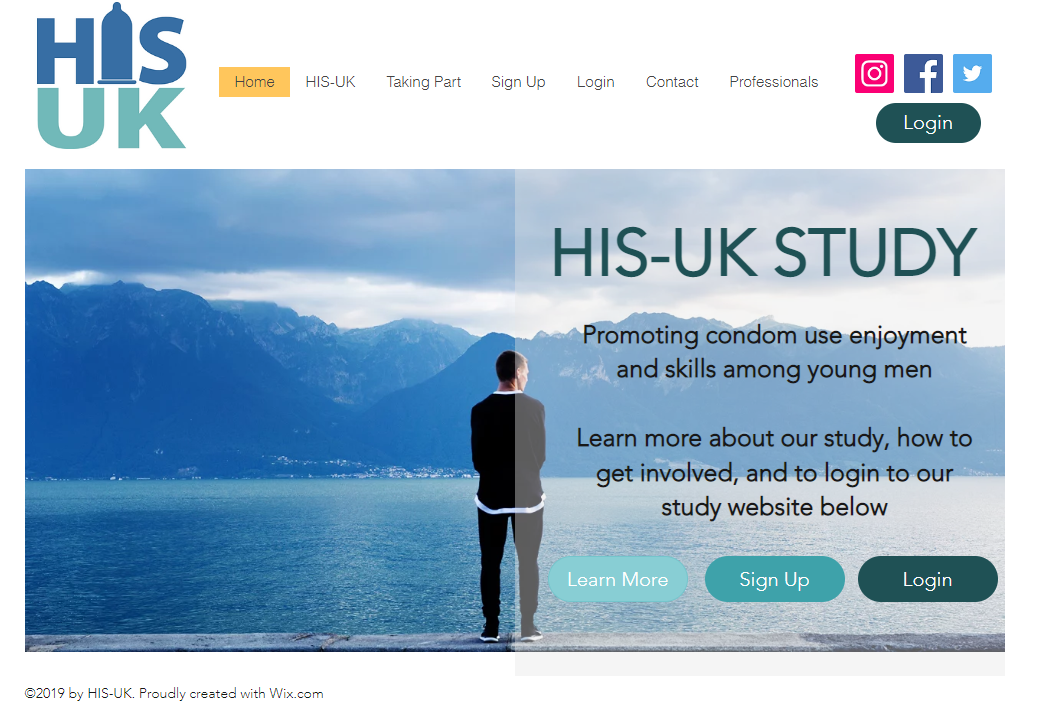
Home-based intervention strategy (HIS-UK) website.

Due to the uncertain future of the COVID-19 pandemic and the need to reduce direct-contact exposure between site staff and participants, the following amendments to the original protocol were proposed and approved by the UK Health Research Authority in February 2021: (1) potential participants can self-refer to the study by completing a web-based expression of interest application via the study website (Figure 3). Eligible participants, who live within the catchment area of a recruiting NHS Trust site, are prompted to complete the web-based consent, *Lifeguide* registration, and T0 baseline questionnaire before being contacted by site staff to complete the final recruitment tasks (verbal reconfirmation of informed consent, chlamydia screening, and delivery of the intervention arm); (2) participants randomized to the control arm or to e-HIS are not required to attend in person to a recruitment site to complete the final recruitment tasks; these instead can be performed during a telephone consultation. For participants randomized to ProHIS, the recruitment tasks can be performed either using the web-based video-consultation software or in clinic. ProHIS cannot be delivered via a telephone consultation due to the requirement of a visual condom demonstration; (3) all HIS-UK condom kits can be mailed out to participants instead of being collected in person; (4) postal STI screening kits can be used to collect and return samples for chlamydia screening; and (5) the number of NHS Trust sites recruiting to the trial has been increased, with no upper limit proposed.

### HIS-UK Tasks and Data Collection

Following education and training, HIS-UK participants commence a 2-week condom and lubricant self-practice period using the contents of the supplied kits and guided home-based exercises (ie, applying, using, and removing condoms without partner presence). As young men try out each condom and lubricant, they are asked to think about what they like and dislike about the products (texture, smell, thickness, shape, size, etc), and to focus on pleasurable sensations when using them to build positive associations between condom use and sexual enjoyment. After experimentation, participants are asked to complete a web-based rating and reflection form for each condom and lubricant used.

Automated emails or SMS text message reminders are sent to participants to complete their ratings. Those who complete at least three ratings over the 2 weeks can order 12 condoms of their choosing and 6 sachets of lubricant to receive by post.

### Follow-up

All participants receive monthly automated text message or email notifications to complete a web-based questionnaire (T1-T12), as per T0.

HIS-UK participants who successfully complete a follow-up questionnaire, and who report sexual activity within the previous month, are offered the opportunity to order further supplies of condoms and lubricants of their choosing (12 condoms and 6 lubricants).

At T6 and T12, all trial participants are requested to provide samples for chlamydia testing and are offered the choice of using a postal screening kit or attending at their trial registration clinic. As per usual clinical practice, only participants reporting sexual activity in the previous 6 months are screened. Furthermore, if a participant has reported chlamydia screening within 4 weeks prior to the T6 and T12 testing period, the self-reported results of these tests are used, and no further samples are taken to minimize screening burden.

All participants receive electronic voucher payments totaling £50 (US $60) to compensate them for their time; £10 (US $12) after active participation for 3 months, £15 (US $18) after 6 months, and £25 (US $30) after 12 months.

### Primary Outcome Measure

Chlamydia test positivity is measured at baseline (T0), 6 months (T6), and 12 months (T12) through biomarker testing and treatment, and at T1-T5 and T7-T11 through questionnaire self-report. The primary health end point is test positivity at 6 months. To examine longevity of intervention effect, test positivity is assessed again at 12 months after randomization.

### Secondary Process Indicators

The following secondary process indicators (those through which the primary outcome is likely to be realized) are measured via web-based self-completion questionnaires using validated scales at baseline (T0) and at monthly intervals to 12 months (T1-T12)—the *Condom Barriers Scale* [27], with items including the effect of condoms on sexual experience (eg, “condoms reduce orgasm/climax”) and motivational barriers (eg, “I feel closer to my partner without a condom”); the *Condom Use Errors and Problems Survey* [28,29] to assess the possible errors (eg, putting condom on after starting sex) and problems (eg, breakage or slippage and erection difficulties) when using condoms; the *Correct Condom Use Self-Efficacy Scale* [30] to measure the efficacy of an individual to negotiate and correctly use condoms with their partner; the *UCLA Multidimensional Condom Attitude Scale* [31], in which items include “condoms can make sex more pleasurable/stimulating,” “use of condoms can improve foreplay,” “condoms can feel good for both partners,” and “condoms are fun”; and finally, the *Condom Fit and Feel Scale* [32], in which participants are asked to recall their recent condom use experiences and answer questions about fit and feel.

Additionally, participants are asked at baseline and monthly intervals about their sexual partners, frequency of intercourse, STI screening and test positivity, condom use motivation, use of contraception and condoms, and any episodes of condomless anal or vaginal intercourse.

### Economic Evaluation—Outcome Data

Alongside the clinical outcomes collected in the trial and in line with guidance by the National Institute for Health and Care Excellence, quality-adjusted life years will be used as an outcome measure to assess the cost-effectiveness of HIS-UK as compared to usual condom distribution care [33]. Because sexual health interventions such as HIS-UK have an important psychosocial aspect, the following 2 validated measures of health-related quality of life, which ask about an individual’s self-perceived physical and psychological health status, are used: the SF-12 (12-Item Short Form Health Survey) instrument and the EQ-5D-5L questionnaire [34-36]. These questionnaires are administered to participants to compare changes in HRQL for the 3 trial arms, at baseline, 6 months, and 12 months after randomization.

### Cost and Resource Use Data

Resource use data will be collected prospectively to estimate the costs associated with the 2 HIS-UK intervention arms, compared to usual care. Within the trial, the resource use and costs associated with delivering ProHIS and e-HIS and any follow-up care will be captured via trial reporting mechanisms. This will include the costs of condom kits, consultation costs, digital delivery costs, and other resource use associated with intervention delivery. The baseline and monthly questionnaires (T0-T12) further capture the wider NHS and public sector resource use by participants, including the use of medication, general practitioner and sexual health service visits, and other public sector resource usage. The monthly questionnaires also collect data on personal costs experienced by participants connected with their involvement in the trial (eg, travel costs, internet use, and other out-of-pocket expenses).

### Sample Size

The clinical effectiveness of HIS-UK delivered by ProHIS and e-HIS will be analyzed with an overall Type I error rate of 5% (2.5% per comparison), comparing test positivity in each of the intervention arms with the control arm (usual care). Data published by the National Chlamydia Screening Programme suggest a test positivity of 11.9% in 2017 and 12.2% in 2018 among young men aged 15-24 years in England tested in specialist and nonspecialist (including community-based) services [37]. Our trial is powered to detect a 45% reduction in chlamydia test positivity among our intervention arms—from 11% to 6% at 6 months after randomization. Previous piloting suggests that the intervention is likely to be equally effective across all stratification subgroups (deprivation, ethnicity, sexual orientation, and age) [22,23].

To have 85% power to obtain the projected difference in the outcome at T6, the study requires 476 participants in each of the arms (G*Power 3.1.9.2). To minimize risk to the trial and to reflect 36% attrition at follow-up (observed during feasibility testing) [22], a total of 2231 participants will be randomized.

It is estimated that it will take 30 months to recruit the target sample of 2231 young men, based on a recruitment rate of 15 per month, per NHS Trust site, using a phased recruitment strategy.

### Process Evaluation and Trial Progression

The first 135 young men recruited and followed up for 6 months will form our internal pilot to assess trial implementation, participant responsiveness, intervention fidelity, and the acceptability of randomization and chlamydia screening for trial continuation. During the assessment, the following questions will be answered: “Can young men be recruited at a reasonable rate and to the numbers anticipated?” “Are young men willing to be randomized within the trial?” “Is chlamydia screening at T0 and T6 sufficiently acceptable and feasible to implement?” “Do young men remain in the study in sufficient numbers at 6-month follow-up?” “Are the intervention and study design sufficiently acceptable?” “Are site staff able to deliver the intervention with reasonable fidelity?”

To assess intervention implementation and engagement, participant access to and usage of e-HIS is recorded along with fidelity of ProHIS intervention delivery and completion of condom ratings. In-depth qualitative interviews with all site staff involved in the recruitment of internal pilot participants will also be conducted to explore acceptability of the research design and ease of trial delivery. Furthermore, at 6 months after randomization, internal pilot participants allocated to the ProHIS and e-HIS trial arms will be invited to participate in interviews to explore study acceptability, issues of contamination and protocol adherence, and intervention benefits. We expect that 20 interviews will be sufficient to reach theoretical saturation; however, if necessary, additional interviews will be undertaken with participants from subsequent recruitment phases.

### Analysis

Analysis and presentation of data will be in accordance with the revised CONSORT 2010 statement [38]. The statistical analysis will be performed on available cases following intention-to-treat principles with due emphasis placed on confidence intervals for the between-arm comparisons. Baseline demographics (eg, age, ethnicity, deprivation, and sexual orientation) and self-reported outcome measure data (secondary process indicators) will be assessed for comparability between the arms using descriptive analyses.

The primary analysis will be undertaken using generalized linear modelling to compare the effectiveness of HIS-UK against usual care in reducing chlamydia test positivity at T6. The analysis will be repeated at T12 to examine longevity of intervention effect. Analyses will be extended to include the investigation of possible intervention moderators and mediators, the exploration of process measures (eg, number of condom ratings completed), and the identification of which young men most benefit from ProHIS and e-HIS (according to age, sexual orientation, ethnicity, and social deprivation). Similar comparative analyses using the secondary process indicators collected at T0-T12 will be undertaken using generalized linear mixed modelling to allow for the analysis of repeated measurements over time and comparison between the study arms. To meet our intention-to-treat principles analysis, withdrawals and protocol violators will be analyzed in their arms as randomized.

To assess the costs and benefits of HIS-UK (delivered via ProHIS and e-HIS) compared with usual care, both a within-trial analysis and a model-based economic analysis will be undertaken. The main within-trial economic analysis will assess cost-effectiveness based on incremental cost per quality adjusted life year gained at 6 months, with a secondary analysis of cost per case of chlamydia avoided at 6 months, reflecting the primary outcome of the trial; this analysis will then be repeated to measure cost-effectiveness over a 12-month period. Initially, the base case analysis will be framed in terms of a cost-consequence analysis for the 3 trial arms, and data will be reported in a disaggregated manner on the incremental cost and important consequences assessed in the trial.

If the trial shows that ProHIS or e-HIS are effective in reducing chlamydia positivity and other condom use health behavior outcomes, compared with usual condom distribution care, there are likely to be important cost implications for the health care sector, the wider public sector, and for society as a whole. If deemed necessary, a decision-analytic model will be used to extrapolate costs and outcomes beyond the end of the trial and synthesize data on costs and outcomes from a range of sources [39]. The evidence used in the model will be drawn from the trial and a comprehensive review of the literature on condom use and failure, prevalence of chlamydia and other STIs, transmission rates, and long-term outcomes. If data availability permits, a public sector and an NHS perspective will be adopted, in line with recommendations [40].

The results will be presented using cost-effectiveness acceptability curves to show the uncertainty surrounding the cost-effectiveness of the ProHIS and e-HIS interventions, for a range of thresholds for cost-effectiveness [41]. Both deterministic and probabilistic sensitivity analyses will be used to explore the inherent uncertainty around the estimates employed in the evaluation [39].

### Ethics Approval

Ethics approval for the randomized controlled trial has been obtained from the University of Southampton Research Integrity and Governance Committee, and the Health Research Authority South Central Oxford B Committee (REC reference: 19/SC/0486).

## Results

Funding was secured in February 2019, and recruitment was commenced in March 2020; however, due to the COVID-19 pandemic, recruitment was halted in April 2020. As a result, the study was adapted to reduce clinical contact between recruitment site staff and participants (see “Post–COVID-19 Amendments”), and subsequently reopened to recruitment in July 2021, with a planned participant recruitment period of 30 months and a 12-month participant follow-up.

## Discussion

### Overview

The hypothesized main findings of this trial are that the HIS-UK intervention (delivered by either ProHIS or e-HIS) will reduce chlamydia test positivity among young men (16-25 years) by enhancing condom use experiences and improving correct and consistent condom use, as compared to usual condom distribution care. In addition, it is anticipated that the HIS-UK intervention, as compared to usual condom distribution care, will be cost-effective.

### Strengths and Limitations

The strengths of this protocol include the use of a randomized controlled trial design, the targeted large sample, and the use of chlamydia biomarker testing. A limitation of the design is the restriction of participants to men aged 16-25 years. Future directions include adapting and evaluating the intervention among women and men of a broader age range.

### Dissemination Plan

The results from this study will be presented at scientific conferences, published in peer-reviewed journals, and shared on social media. We will also share our findings with key stakeholders, including young men, clinicians, and commissioners of sexual health services.

### Conclusions

This is the first randomized controlled trial evaluation of the HIS-UK intervention with young men. If the intervention is effective and cost-effective, this could have a positive impact on NHS services by reducing the incidence of STI rates and relieving pressure on staff time, financial costs, and other resources in the treatment of STIs. The intervention may also encourage sexual health services to adopt additional digital technologies and ultimately improve access to such services for young people, decreasing health inequalities engendered by fear of stigmatization.

## Abbreviations

CUE: condom use experience
e-HIS: HIS-UK delivered digitally
HIS-UK: home-based intervention strategy
NHS: National Health Service
ProHIS: HIS-UK delivered face-to-face
SF-12: 12-Item Short Form Health Survey
STI: sexually transmitted infection
